# Design of a SiC MOSFET Gate Driver Chip Based on Adaptive Active Drive Technology

**DOI:** 10.3390/mi17050558

**Published:** 2026-04-30

**Authors:** Qidong Li, Yuxin Zhang, Baoqiang Huang, Weihua Zhang, Chen Chen, Jianming Lei, Desheng Zhang, Run Min, Qiaoling Tong

**Affiliations:** 1 School of Integrated Circuits, Huazhong University of Science and Technology, Wuhan 430074, China; qidongli@hust.edu.cn (Q.L.); d202281038@hust.edu.cn (Y.Z.); d202387049@hust.edu.cn (B.H.); leijianming@hust.edu.cn (J.L.); dszhangic@hust.edu.cn (D.Z.); minrun@hust.edu.cn (R.M.); tongqiaoling@hust.edu.cn (Q.T.); 2 School of Electronic Information, Wuhan University of Science and Technology, Wuhan 430081, China; chenchen@wust.edu.cn

**Keywords:** SiC MOSFET, active gate driver, cross-cycle regulation, Miller plateau voltage, adaptive control, EMI suppression

## Abstract

Silicon carbide (SiC) MOSFETs are promising for high-efficiency, high-power-density power conversion owing to their high breakdown capability, fast switching speeds, and low switching losses. However, parasitic parameters can cause severe voltage/current overshoot and oscillation during high-speed switching, leading to electromagnetic interference and degraded performance. To address this issue, this study analyzes the mechanisms of current overshoot during turn-on and voltage overshoot during turn-off, and presents an adaptive active gate driver chip based on a three-stage driving current control strategy. By identifying key switching intervals and regulating segmented gate-drive current, the proposed chip can effectively suppress overshoot while reducing the switching loss. During turn-on, cross-cycle switching point regulation based on Miller plateau tracking is proposed to achieve adaptive control under different operating conditions, while the turn-off control is realized by peak sampling of the drain–source voltage. The chip was fabricated in the 180 nm BCD process. Compared with a conventional passive driver, the proposed driver reduces turn-on loss by 35.1% at 400 V/40 A under a d*v*_DS_/d*t* of 4.8 V/ns and reduces turn-off loss by 33.2% under a *v*_DS_ overshoot of nearly 50 V. These results show that the proposed chip improves SiC MOSFET switching performance and provides a practical gate-driving solution.

## 1. Introduction

Against the backdrop of the global energy transition and carbon-neutrality targets, power-conversion systems with high efficiency, high power density, and high reliability have become a major development direction. Wide-bandgap (WBG) semiconductor devices, including silicon carbide (SiC) and gallium nitride (GaN), are continuously pushing power electronic systems toward higher switching frequency, higher efficiency, and higher power density because of their superior material properties. Among them, silicon carbide metal–oxide–semiconductor field-effect transistors (SiC MOSFETs), which feature high breakdown capability, fast switching speed, and low switching loss [1], have been widely used in high-voltage and high-power applications such as new-energy vehicles, power conversion, communication power supplies, and smart grids [2,3].

However, the practical deployment of SiC MOSFETs under high-voltage and high-frequency switching conditions still faces significant challenges. Because of device packaging and circuit layout, parasitic inductances and capacitances inevitably exist in both the power loop and the gate-driving loop. During high-speed switching, these parasitic parameters lead to severe voltage/current overshoot and oscillation under high-d*v*/d*t* and high-d*i*/d*t* conditions, which in turn cause strong electromagnetic interference (EMI) [4,5,6]. In addition, oscillation of the drain–source voltage can be coupled to the gate through the gate–drain capacitance, thereby inducing false turn-on and threatening safe system operation. Therefore, suppressing overshoot and oscillation while preserving the low-loss switching advantage of SiC MOSFETs has become a key issue in their application [4,7,8,9,10].

To address these problems, the available optimization methods mainly include board-level parasitic-parameter optimization [11,12] and gate-driving-strategy optimization. Compared with board-level optimization, which is strongly constrained by physical layout, gate-driving techniques can directly control the switching transient and therefore offer better universality and engineering applicability. Conventional passive gate drivers (PGDs) reduce the switching speed by using a fixed gate resistor. Although this method can suppress overshoot and oscillation to some extent, it also increases switching loss significantly and therefore cannot fully exploit the high-speed, low-loss capability of SiC MOSFETs. Consequently, active gate drivers (AGDs), which dynamically adjust the driving capability during switching to jointly optimize loss, overshoot, and EMI, have become a major research focus [13,14,15,16,17,18,19,20].

From the perspective of control strategy, existing AGD techniques can be broadly classified into closed-loop feedback, open-loop, and feedforward schemes. Closed-loop feedback methods based on current slew rate or voltage slew rate can effectively suppress overshoot; however, they impose extremely stringent requirements on loop speed and analog front-end bandwidth, which increases implementation difficulty and power consumption. In contrast, open-loop and fixed-threshold feedforward methods avoid the challenges of ultra-fast closed-loop design, but they are difficult to adapt to parameter drift caused by load and temperature variations. Therefore, a gate-driving strategy that can balance response speed, implementation complexity, and adaptability to operating conditions still requires further investigation [21,22,23,24,25,26,27].

To address these issues, this paper proposes an active gate-driver chip for SiC MOSFETs based on an adaptive feedforward control technique. In the proposed scheme, the gate–source voltage *v*_GS_ is used as the feedforward signal, and a cross-cycle control architecture tracks the Miller plateau voltage to identify the key switching stages and dynamically regulate the multi-stage gate current. On this basis, the paper introduces the overall architecture of the proposed driver chip and the implementation of its key circuits, and verifies its performance under different DC-bus voltages, load currents, and temperature conditions through simulation and experiment. The results demonstrate that the proposed driving scheme provides an effective approach for suppressing voltage/current overshoot and reducing switching loss in high-frequency, high-power-density SiC MOSFET applications.

The remainder of this paper is organized as follows. Section 2 presents the switching characteristics of the SiC MOSFET and the operating principle of the proposed gate-driving strategy. Section 3 describes the overall architecture of the proposed gate-driver chip and the implementation of the key circuits. Section 4 presents the simulation results, test platform, experimental results, and comparative analysis. Finally, Section 5 summarizes the main work and presents the conclusions.

## 2. Switching Characteristics and Adaptive Gate-Driving Strategy

**Figure 1:**
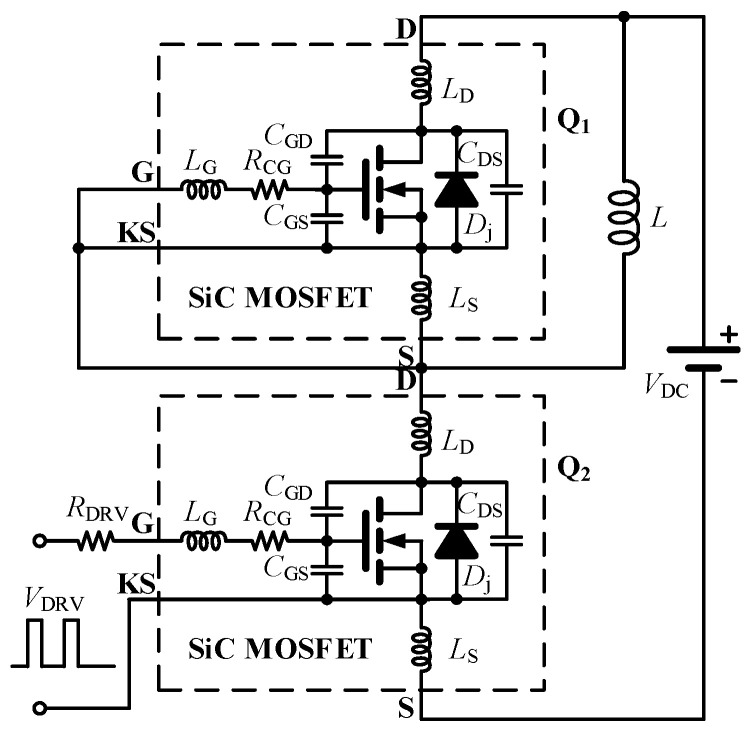
Double pulse test circuit constructed from the equivalent parasitic parameter model of the SiC MOSFET.

In this section, the switching behavior of the SiC MOSFET is analyzed in detail. By considering the coupling relationships among gate-charge transfer, drain–source voltage, and drain current during the switching transient, analytical models for both the turn-on and turn-off processes are established. On this basis, an adaptive three-stage driving scheme for turn-on control and a three-stage feedback driving scheme for turn-off control are proposed according to the dynamic characteristics of different switching stages, so as to achieve optimized regulation of voltage/current overshoot and switching loss. The following analysis of switching overshoot is carried out based on the Double Pulse Test (DPT) circuit shown in Figure 1.

### 2.1. SiC MOSFET Turn-On Process Analysis and the Proposed Three-Stage Driving Scheme for Turn-On Control

**Figure 2:**
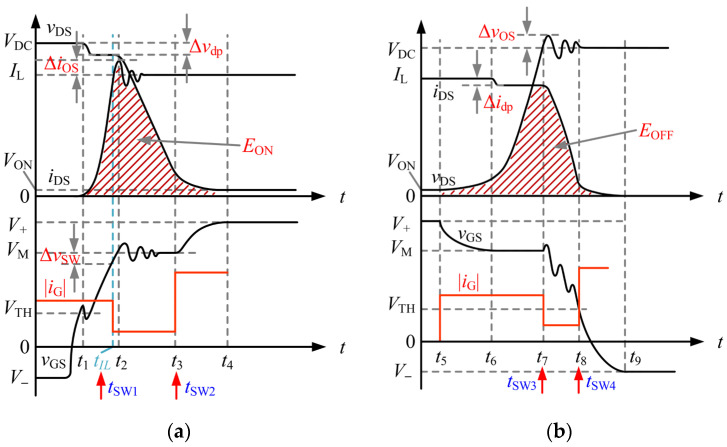
(**a**) Modeling of the turn-on process and the proposed three-stage control scheme for turn-on control. (**b**) Modeling of the turn-off process and the proposed three-stage control scheme for turn-off control.

According to the changes in gate–source voltage *v*_GS_, drain current *i*_DS_, and the drain–source voltage *v*_DS_, the turn-on transient process of the SiC MOSFET can be divided into four stages: the turn-on delay stage, current rising stage, voltage falling stage, and gate recharging stage. The total duration of these four stages can be defined as the turn-on transient time. The typical theoretical waveforms of this process are illustrated in Figure 2a. In the following, these four stages are analyzed individually based on the theoretical waveforms.

(1) Turn-On Delay Stage (0–*t*_1_)

The driver output transitions from the minimum voltage *V*_−_ to the maximum voltage *V*_+_, and the gate-driving current charges the input capacitance *C*_ISS_ ≈ *C*_GS_ through the gate resistance *R*_G_ = *R*_DRV_ + *R*_CG_. During this interval, *v*_GS_ has not yet reached the threshold voltage *V*_TH_, and the device therefore remains in the fully off state. Accordingly, the drain current *i*_DS_ is 0, while the drain–source voltage remains as the DC bus voltage *V*_DC_. Since *i*_DS_ = 0 in this stage, no switching overlap loss is generated. However, a small driving current during this stage results in a large turn-on delay. Neglecting the variation in *C*_ISS_, the duration of this stage can be approximately written as

(1) $$t_{d,\mathrm{on}}\approx \frac{C_{\mathrm{ISS}}(V_{\mathrm{TH}}-V_{-})}{I_{G1}}$$

Equation (1) indicates that increasing the initial gate current can shorten the turn-on delay. Because the power-current commutation has not yet started in this interval, the contribution of this stage to EMI is small and can be neglected.

(2) Current Rising Stage (*t*_1_*–t*_2_)

Once the gate–source voltage exceeds *V*_TH_, the channel is formed, and the drain current *i*_DS_ rises rapidly according to the device transconductance characteristic. Meanwhile, the load current begins to commutate from the freewheeling diode of the upper switch to the main switching device. During this interval, the large d*i*_DS_/d*t* causes an inductive drop in *v*_DS_ due to the parasitic inductance, which can be expressed as Equation (2):

(2) $$\Delta v_{\mathrm{dp}}=L_{p}\frac{di_{DS}}{dt}=(L_{D2}+L_{S2})\frac{di_{DS}}{dt}.$$

More critically, the extremely high d*i*/d*t* excites the reverse-recovery behavior of the upper-switch diode, generating the reverse-recovery charge *Q*_rr_ and thereby inducing an overshoot current Δ*i*_DS1_.

(3) $$\Delta i_{DS1}=\sqrt{\frac{2Q_{\mathrm{rr}}}{S+1}\cdot \frac{di_{DS}}{dt}}.$$

At the same time, the change in *v*_DS_ injects a displacement current Δ*i*_DS2_ through the parasitic capacitances. The superposition of these two components gives rise to the total current overshoot, i.e., Δ*i*_os_ ≈ Δ*i*_DS1_ + Δ*i*_DS2_. Evidently, the drain-current slew rate d*i*_DS_/d*t* in this stage is strongly governed by the transient gate current *i*_G_.

To further clarify the correlation among gate current, switching loss, and EMI in this stage, the current-rising interval can be approximately expressed from the gate-charge viewpoint as

(4) $$t_{I}\approx \frac{Q_{GS1}}{I_{G1}}.$$

where *Q*_GS1_ is the gate charge required to raise the channel current from zero to the load-current level. Since the channel current of the SiC MOSFET follows the transfer characteristic $i_{\mathrm{ch}}=K_{n}{\left(v_{GS}-V_{\mathrm{TH}}\right)}^{2}$, a larger *I*_G1_ leads to a faster rise in *v*_GS_ and thus a larger current slew rate. Therefore, the current slew rate can be approximately summarized as

(5) $$\frac{di_{DS}}{dt}\approx g_{m}\frac{dv_{GS}}{dt}=g_{m}\frac{I_{G1}}{C_{\mathrm{ISS}}}.$$

Accordingly, the overlap loss in this stage can be estimated as

(6) $$E_{\mathrm{on},I}\approx \frac{1}{2}V_{\mathrm{DC}}I_{L}t_{I}\approx \frac{V_{\mathrm{DC}}I_{L}Q_{GS1}}{2I_{G1}}.$$

Equations (4)–(6) indicate that increasing *I*_G1_ shortens the current commutation time and reduces the overlap loss in the early turn-on interval, but it also increases the reverse-recovery current of the complementary diode, the current overshoot caused by parasitic inductances, and the conducted/radiated EMI associated with the high-d*i*/d*t* current loop. Therefore, the first-stage gate current should be selected as a compromise between switching speed and current-overshoot suppression.

(3) Voltage Falling Stage (*t*_2_–*t*_3_)

When *i*_DS_ reaches and exceeds the actual load current *I*_L_, *v*_GS_ is clamped at the Miller plateau voltage *V*_M_. At this point, the gate charge is no longer used to increase the channel current; instead, it is entirely utilized to remove charge from *C*_GD_, thereby causing *v*_DS_ to fall rapidly to the on-state voltage drop. The falling rate of *v*_DS_ is strictly governed by the gate current *i*_G_ injected during this stage:

(7) $$\left|\frac{dv_{DS}}{dt}\right|=\frac{i_{G}}{C_{\mathrm{GD}}}=\frac{V_{+}-V_{M}}{R_{G}C_{\mathrm{GD}}}.$$

Since the power loop is in a low-impedance underdamped state at this moment, an excessively high d*v*_DS_/d*t* will result in severe voltage ringing and electromagnetic interference (EMI). This stage also corresponds to the interval with the largest overlap between voltage and current, and therefore contributes the majority of the turn-on loss *E*_ON_.

To make the above relationship more explicit, the duration of the voltage-falling stage can be approximately written as

(8) $$t_{\mathrm{II}}\approx \frac{Q_{\mathrm{GD}}}{I_{G2}}.$$

where *Q*_GD_ is the Miller charge. Accordingly, the turn-on energy consumed in this stage can be approximated as

(9) $$E_{\mathrm{on},\mathrm{II}}\approx \frac{1}{2}V_{\mathrm{DC}}I_{L}t_{\mathrm{II}}\approx \frac{V_{\mathrm{DC}}I_{L}Q_{\mathrm{GD}}}{2I_{G2}}.$$

Equations (7)–(9) indicate that *I*_G2_ directly determines the trade-off between switching loss and EMI during the Miller interval. Increasing *I*_G2_ shortens the voltage-falling duration and reduces the dominant part of the turn-on loss, but it also leads to a larger d*v*_DS_/d*t*, stronger displacement current through parasitic capacitances, more severe voltage ringing, and higher EMI. Therefore, the second-stage gate current should be intentionally reduced to suppress d*v*_DS_/d*t* and overshoot-related EMI, while accepting a moderate increase in turn-on loss.

(4) Gate Recharging Stage (*t*_3_–*t*_4_)

After *v*_DS_ decreases to nearly zero, the device leaves the saturation region and enters the linear region, while *v*_GS_ continues to rise from *V*_M_ to the target high level *V*_+_. The primary objective of this stage is to reduce *R*_DS(on)_ as rapidly as possible. The duration of this stage can be approximately expressed as

(10) $$t_{\mathrm{III}}\approx \frac{Q_{GS2}}{I_{G3}}.$$

where *Q*_GS2_ denotes the remaining gate charge after the Miller interval. Since the overlap between *v*_DS_ and *i*_DS_ is already very small in this stage, its contribution to switching loss and EMI is limited. Therefore, a relatively large *I*_G3_ is preferred to rapidly reduce *R*_DS(on)_ and shorten the residual turn-on time.

Based on the theoretical analysis of the switching dynamics, this study proposes a three-stage turn-on driving strategy. In Figure 2a, *t*_SW1_ and *t*_SW2_ denote the switching instants of the gate-driving current. The first switching point is defined at the critical instant *t*_SW1_ = *t*_IL_, and the second switching point is set at *t*_SW2_ = *t*_3_.

Based on *t*_SW1_ and *t*_SW2_, a three-stage driving current is provided during the turn-on process.

- Stage I (0–*t*_IL_): This stage starts from the turn-on trigger instant and ends when the drain current *i*_DS_ rises to the load current *I*_L_, which includes the turn-on delay stage and the current rising stage. During this interval, the driver provides a constant gate current *I*_G1_ with a moderate driving capability, so as to prevent an excessively large d*i*_DS_/d*t*, which could otherwise increase the peak value of the diode reverse-recovery current.
- Stage II (*t*_IL_–*t*_3_): Once *i*_DS_ reaches *I*_L_ and *v*_GS_ enters the Miller plateau *V*_M_, the driver promptly reduces the output current to a much lower level, denoted by *I*_G2_. This stage fully covers the voltage falling interval. Owing to the reduced gate current, d*v*_DS_/d*t* is effectively limited, which suppresses the displacement current coupled through parasitic capacitances and, consequently, mitigates current overshoot Δ*i*_DS_ and voltage ringing.
- Stage III (*t*_3_–*t*_4_): After *v*_DS_ falls to the conduction voltage and the device enters the linear region, *v*_GS_ leaves the Miller plateau and starts to rise again. At this point, the driver delivers the maximum peak current *I*_G3_ to raise the gate voltage to *V*_+_ as rapidly as possible.

**Figure 3:**
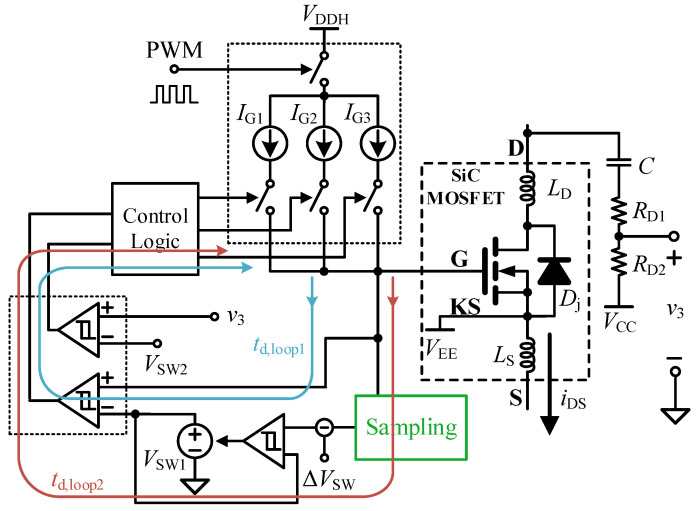
Turn-on control scheme based on cross-cycle feedforward.

To improve the adaptability of the three-stage driving scheme to parameter drift and operating-condition variations, a cross-cycle feedforward adaptive mechanism based on Miller plateau tracking is further introduced. As illustrated in the control scheme shown in Figure 3, the three current sources *I*_G1_, *I*_G2_, and *I*_G3_ represent the gate-driving current levels for Stage I, Stage II, and Stage III of the turn-on process, respectively. They are not applied simultaneously. Instead, the control logic selects them sequentially according to the switching thresholds *V*_SW1_ and *V*_SW2_, so that only one current source is active in each stage. During the current switching cycle, the control system captures the actual Miller plateau voltage *V*_M_, which reflects the load current *I*_L_ and temperature characteristics, through a sampling circuit. By combining this information with a preset compensation term (Δ*V*_SW_), which accounts for circuit delay and the voltage drop across the gate resistance *R*_G_, the trigger threshold *V*_SW1_ for the transition from the first stage to the second stage in the next switching cycle is generated by the control logic through cross-cycle regulation. In this way, when the load current or operating temperature changes, the corresponding shift in *V*_M_ can be tracked in the current cycle and used to update *V*_SW1_ in the next cycle, so that the Stage-I/Stage-II switching point is adaptively adjusted under different operating conditions. By allocating the voltage comparison and regulation to the idle interval of the switching cycle, this cross-cycle method can effectively accommodate variations in load conditions and wide-temperature drift, while also reducing the bandwidth requirement and power consumption of the analog front-end for ultrafast response.

### 2.2. SiC MOSFET Turn-Off Process Analysis and the Proposed Three-Stage Driving Scheme for Turn-Off Control

The turn-off process can be divided into four stages, namely the gate discharging stage, voltage rising stage, current falling stage, and turn-off delay stage. Correspondingly, the total duration of these four stages is defined as the turn-off transient time. The classical theoretical waveforms of this process are shown in Figure 2b. In the following, these four stages are analyzed individually based on the theoretical waveforms.

(1) Gate Discharging Stage (*t*_5_–*t*_6_)

As the driver output steps from the maximum driving voltage *V*_+_ to the minimum driving voltage *V*_−_, the gate–source voltage *v*_GS_ begins to decrease from *V*_+_. During this stage, the drain current *i*_DS_ remains equal to the load current *I*_L_, while *R*_DS(on)_ gradually increases, causing *v*_DS_ to rise and thereby introducing additional switching loss. Neglecting the nonlinear variation in *C*_ISS_, the duration of this pre-discharge interval can be approximately expressed as

(11) $$t_{d,\mathrm{off}}\approx \frac{C_{\mathrm{ISS}}(V_{+}-V_{M})}{I_{G1}}.$$

Since this interval mainly prepares the device to enter the Miller plateau, its effect on EMI is weaker than that of the following two stages.

(2) Voltage Rising Stage (*t*_6_–*t*_7_)

When the gate–source voltage *v*_GS_ decreases to the Miller plateau voltage *V*_M_, the SiC MOSFET changes from the linear region to the saturation region, and the turn-off process enters the voltage rising stage. During this stage, the drain–source voltage *v*_DS_ continues to rise, but it is still insufficient to turn on the parasitic body diode of the upper switch Q_1_; therefore, the drain current *i*_DS_ remains equal to the load current *I*_L_. Meanwhile, *v*_GS_ is clamped at the Miller plateau voltage throughout this stage, indicating that the gate current *i*_G_ is entirely used to discharge the gate–drain capacitance *C*_GD_. Because the voltage difference between the minimum driving voltage *V*_−_ and *V*_M_ is larger than that between *V*_+_ and *V*_M_, the resulting d*v*_DS_/d*t* during turn-off is higher than that of turn-on. In addition, as *v*_DS_ increases, the nonlinear capacitance *C*_GD_ gradually decreases, which further contributes to a larger d*v*_DS_/d*t*.

Moreover, the relatively large d*v*_DS_/d*t* in this stage gives rise to displacement currents in the drain–source capacitance *C*_DS_ of Q_2_ and the parallel parasitic capacitance *C*_F_ of Q_1_, causing *i*_DS_ to decrease slightly. The displacement current Δ*i*_dp_ can be expressed as follows:

(12) $$\Delta i_{\mathrm{dp}}=(C_{F}+C_{DS})\frac{dv_{DS}}{dt}=\frac{\left(V_{M}-V_{-})(C_{F}+C_{DS}\right)}{R_{G}C_{\mathrm{GD}}}.$$

To further establish the relationship among gate current, switching loss, and EMI in this stage, the voltage-rising interval can be approximately expressed as

(13) $$t_{I,\mathrm{off}}\approx \frac{Q_{\mathrm{GD}}}{I_{G1}}.$$

and the corresponding overlap loss can be estimated as

(14) $$E_{\mathrm{off},I}\approx \frac{1}{2}V_{\mathrm{DC}}I_{L}t_{I,\mathrm{off}}\approx \frac{V_{\mathrm{DC}}I_{L}Q_{\mathrm{GD}}}{2I_{G1}}.$$

Equations (12)–(14) indicate that increasing *I*_G1_ shortens the voltage-rising interval and reduces the corresponding overlap loss, but it also aggravates displacement current, voltage ringing, and EMI because of the larger d*v*_DS_/d*t*. Therefore, a moderate sinking current should be used in this stage to balance turn-off speed and d*v*_DS_/d*t*-induced EMI.

(3) Current Falling Stage (*t*_7_–*t*_8_)

After the drain–source voltage *v*_DS_ rises to the DC bus voltage *V*_DC_, the turn-off process enters the current falling stage. In this stage, *v*_GS_ continues to decrease from the Miller plateau voltage *V*_M_, and the drain current *i*_DS_ of Q_2_ begins to decrease continuously with the further reduction in *v*_GS_; accordingly, the parasitic body diode of Q_1_ starts to conduct in the reverse direction. When *v*_GS_ decreases to *V*_TH_, Q_2_ is completely turned off, and its drain current commutates to the upper body diode, marking the end of the current falling stage. In this stage, the high d*i*_DS_/d*t* together with the stray parasitic inductance will lead to overshoot in *v*_DS_. The voltage overshoot Δ*v*_OS_ can be expressed as follows:

(15) $$\Delta v_{\mathrm{OS}}=(L_{D2}+L_{S2})\frac{di_{DS}}{dt}.$$

To further clarify the role of gate current in this interval, the current-falling duration can be approximately written as

(16) $$t_{\mathrm{II},\mathrm{off}}\approx \frac{Q_{GS2,\mathrm{off}}}{I_{G2}}.$$

where *Q*_GS2,off_ denotes the effective gate charge corresponding to the current-decay interval. Since the current fall is driven by gate discharge in this stage, the current slew rate can be approximately summarized as

(17) $$\frac{di_{DS}}{dt}\approx g_{m}\frac{dv_{GS}}{dt}=g_{m}\frac{I_{G2}}{C_{\mathrm{ISS}}}.$$

Accordingly, the overlap loss in this stage can be estimated as

(18) $$E_{\mathrm{off},\mathrm{II}}\approx \frac{1}{2}V_{\mathrm{DC}}I_{L}t_{\mathrm{II},\mathrm{off}}\approx \frac{V_{\mathrm{DC}}I_{L}Q_{GS,\mathrm{off}}}{2I_{G2}}.$$

Equations (15)–(18) show that increasing *I*_G2_ accelerates the current decay and reduces the current-falling loss, but it also increases d*i*_DS_/d*t*, thereby resulting in a larger voltage overshoot and stronger EMI. Therefore, a relatively small *I*_G2_ is preferred in this stage to suppress Δ*v*_OS_ and improve turn-off robustness.

(4) Turn-Off Delay Stage (*t*_8_–*t*_9_)

When *v*_GS_ decreases to *V*_TH_, device Q_2_ is completely turned off, and *i*_DS_ decreases to zero, after which the turn-off process enters the delay stage. At this moment, *v*_DS_ remains stable at the DC bus voltage *V*_DC_, and no switching loss is generated. The gate–source voltage *v*_GS_ continues to decrease to the minimum driving voltage *V*_−_. The duration of this stage can be approximately written as

(19) $$t_{\mathrm{III},\mathrm{off}}\approx \frac{Q_{\mathrm{rem}}}{I_{G3}}.$$

where *Q*_rem_ denotes the remaining gate charge after current commutation. Since the overlap between *v*_DS_ and *i*_DS_ is already very small in this stage, its contribution to switching loss and EMI is limited. Therefore, a relatively large *I*_G3_ can be used to rapidly pull down the gate voltage and ensure reliable turn-off.

Compared with the turn-on process, the dynamic switching loss during turn-off is typically lower than that during turn-on. Therefore, a conservative three-stage strategy is adopted for the turn-off process, as shown in Figure 2b. In Figure 2b, *t*_SW3_ and *t*_SW4_ represent the switching instants of the gate-driving current. The system defines the critical point *t*_SW3_ = *t_7_* as the first switching point and *t*_SW4_ = *t*_8_ as the second switching point.

Similarly, a three-stage driving current is provided during the turn-off process.

- Stage I (*t*_5_–*t*_7_): The gate discharging stage and the voltage rising stage, during which a high d*v*_DS_/d*t* is likely to occur, are combined into the first stage, in which a moderate sinking current *I*_G1_ is applied.
- Stage II (*t*_7_–*t*_8_): During the current falling stage, in which voltage overshoot Δ*v*_OS_ is likely to be induced, the driving current is switched to a smaller sinking current *I*_G2_ to discharge the gate charge more slowly, thereby suppressing d*i*_DS_/d*t* and reducing Δ*v*_OS_.
- Stage III (*t*_8_–*t*_9_): During the turn-off delay stage, the driving current is switched to a larger sinking current *I*_G3_ to rapidly pull down the gate voltage and ensure reliable turn-off.

**Figure 4:**
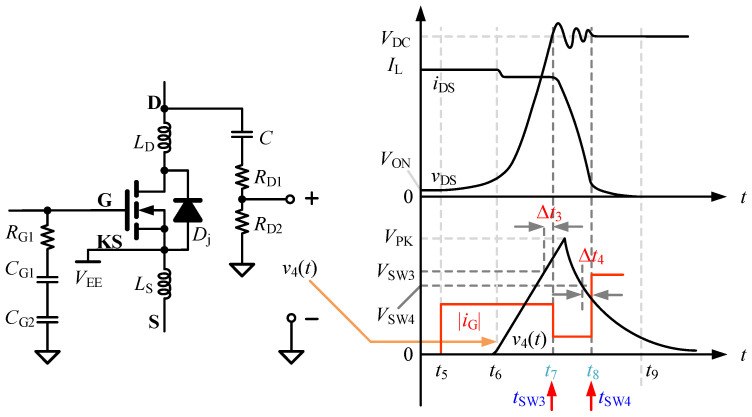
Feedback scheme for the turn-off process.

To improve the robustness of the turn-off process against DC bus fluctuation, parasitic-parameter variation, and load-condition variation, a feedback regulation mechanism is further introduced on the basis of the three-stage turn-off control to monitor key variables in real time and dynamically adjust the turn-off driving strength. During the turn-off process, since *v*_GS_ is already located at the Miller plateau during the voltage rising interval, the *v*_GS_-based feedback strategy adopted in the turn-on process cannot be used. On the other hand, according to the preceding analysis, the optimization requirement for the turn-off process is less strict. Therefore, the three-stage control of the turn-off process is implemented entirely through feedback of the divided *v*_DS_ signal, as shown in Figure 4. In this figure, *R*_D1_ and *R*_D2_ form the drain-voltage divider that generates the low-voltage feedback waveform *v*_4_(*t*), whereas *R*_G1_-*C*_G1_-*C*_G2_ represent the auxiliary gate-side capacitive divider branch retained in the simplified model. In the practical implementation, this capacitive network is used to scale the gate-voltage waveform from approximately −4 V to 15 V down to within the 0–5 V range, so as to facilitate the design of the on-chip sampling circuit. The feedback control strategy for the turn-off process can be summarized as follows: in the current switching cycle, the peak value *V*_PK_ of the divided *v*_DS_ signal is sampled. Based on the result, *t*_SW3_ and *t*_SW4_ are obtained by comparing *V*_PK_ with *V*_SW3_ and *V*_SW4_.

## 3. Overall Architecture and Key Circuit Implementation of the Proposed Driver Chip

### 3.1. Overall Architecture of the Driver Chip

**Figure 5:**
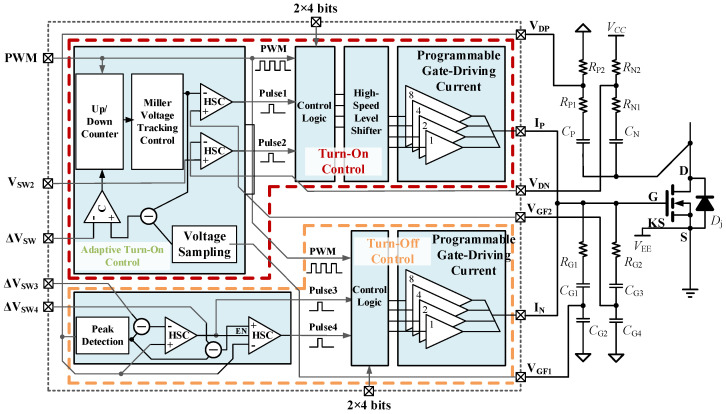
Overall architecture of the proposed adaptive active gate driver chip for the SiC MOSFET.

Based on the preceding analysis of the control strategies and feedback schemes for the turn-on and turn-off processes, an overall architecture of the proposed adaptive active driving scheme for the SiC MOSFET is presented, as shown in Figure 5.

In Figure 5, the black dashed box indicates the major circuit modules integrated on chip, where the red and orange dashed boxes correspond to the modules related to the adaptive turn-on control and the turn-off control, respectively. The adaptive turn-on control unit mainly consists of the Miller plateau voltage sampling circuit, the up/down counter, the Miller plateau voltage tracking control module, and high-speed comparators. Among them, the Miller plateau voltage tracking control module is essentially a digitally controlled voltage source governed by the up/down counter and is used to generate the adaptive switching-point voltage *V*_SW1_.

When the PWM signal transitions from low to high, the driver chip performs turn-on control. At this moment, the adaptive control unit generates the staged trigger signals Pulse1 and Pulse2 through the high-speed comparators. Based on these signals, the control logic module divides the turn-on process into three stages and outputs the corresponding control signals for the driving current of each stage. The driving currents in the first two stages are preset externally, whereas the third stage adopts the maximum driving capability. The tunable gate-driving current module performs staged current switching according to the output of the control logic, thereby generating a three-stage gate-driving current profile. Considering that the on-chip analog modules and the power-driving module operate at different process voltage levels, they are interconnected through a high-speed level-shifting circuit.

The turn-off control module mainly consists of a peak-detection circuit and high-speed comparators. When the PWM signal transitions from high to low, the chip enters the turn-off control process. The peak-detection module samples the peak value of the divided *v*_DS_ feedback signal in the current cycle and, based on this value, establishes the turn-off switching thresholds for the next cycle. During the turn-off stage of the next switching cycle, the high-speed comparators generate the control signals Pulse3 and Pulse4. Subsequently, the turn-off control logic and the driving module combine the PWM signal with the staged signals to generate the three-stage gate-driving current for the turn-off process.

### 3.2. Adaptive Turn-On Control and Gate-Voltage Sensing Circuit

Accurately and stably capturing the subtly varying Miller plateau voltage *V*_M_ in a complex electromagnetic environment is the key challenge in realizing adaptive control, which is achieved by an improved Miller plateau voltage sampling circuit and Miller plateau voltage tracking control circuit.

#### 3.2.1. Improved Miller Plateau Voltage Sampling Circuit

**Figure 6:**
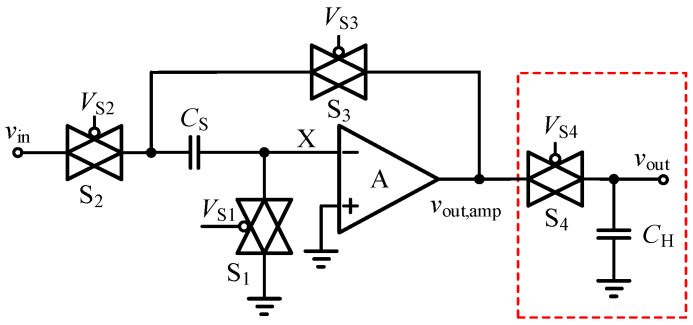
Miller plateau voltage sampling circuit.

Considering that the cross-cycle control only requires a stable voltage to be obtained at the end of each cycle, rather than real-time closed-loop tracking during the transient process, an improved Miller plateau voltage sampling circuit is proposed in this design, as shown in Figure 6.

In the sampling mode, the input-side transmission gates S_1_ and S_2_ are turned on, and the small on-chip sampling capacitor C_S_ directly follows the external RC voltage-divider network in an open-loop, passive, and delay-free manner, while the operational amplifier remains in a bypassed and isolated state. When the sampling trigger signal arrives, i.e., at the instant when the control switches from the second stage to the third stage, S_1_ and S_2_ are rapidly turned off.

At the moment of turn-off, the release of channel charge from the transmission gates injects erroneous charge into *C*_S_, thereby introducing a sampling error Δ*V*_ERR_. The physical model indicates that

(20) $$\Delta V_{\mathrm{ERR}}=\frac{Q_{S2}+Q_{S3}-Q_{S1}}{C_{S}}.$$

To completely eliminate this error, an asymmetric turn-off sequence is adopted in the design, in which S_1_ is turned off before S_2_, and a dummy switch, whose size is precisely set to one half of that of the main switch, is connected in parallel with the critical sensitive node (node X) for charge cancellation. Under this mechanism, the injected electrons are fully absorbed by the parasitic channel of the dummy switch. During the subsequent hold interval, the operational amplifier serves only as a unity-gain buffer with high input impedance to buffer the constant DC voltage stored on *C*_S_ to the subsequent logic stage, thereby completely eliminating the dependence on an ultra-high-gain-bandwidth operational amplifier.

#### 3.2.2. Miller Plateau Voltage Tracking Control Circuit

**Figure 7:**
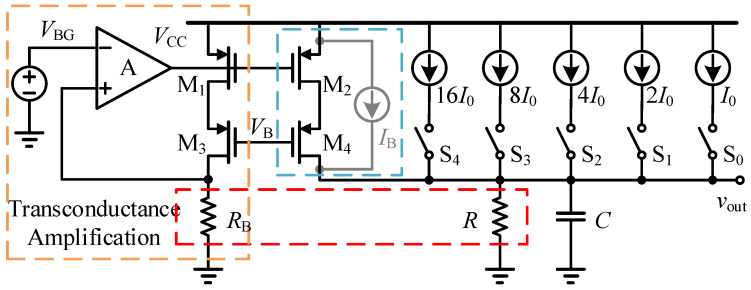
Miller plateau voltage tracking circuit based on the bandgap reference voltage.

The core module of the cross-cycle control is required to generate a high-precision digitally controlled threshold *V*_SW1_ according to the sampled *V*_M_ from the previous cycle. To this end, a current-mode 5-bit digitally controlled Miller plateau voltage tracking circuit based on a Bandgap Reference (BGR) is developed in this work, as shown in Figure 7. With this circuit, the sampled Miller plateau information can be converted into a stable and programmable threshold for the subsequent cycle, thereby improving the robustness of the adaptive turn-on control under load-current and temperature variations.

The core of this circuit relies on the virtual short-circuit and virtual open-circuit characteristics of the operational amplifier, together with a high-precision bandgap reference voltage with temperature-drift compensation, to force an absolutely constant reference current *I*_REF_ through an on-chip precision polysilicon resistor. Subsequently, a large-area cascode current-mirror structure with cross-matched layout is employed to mirror multiple current branches with strictly binary-weighted ratios. The 5-bit digital signal generated by the up/down counter controls the conduction states of the MOS switch network, such that the corresponding weighted currents are summed onto the load reference resistor R, thereby generating a reference voltage *v*_out_ that is insensitive to supply fluctuation:

(21) $$v_{\mathrm{out}}=(N\frac{V_{\mathrm{BG}}}{R_{B}}+S[4:0]I_{0})R.$$

### 3.3. Logic and Auxiliary Functional Circuits

#### 3.3.1. High-Speed Level-Shifting Circuit

**Figure 8:**
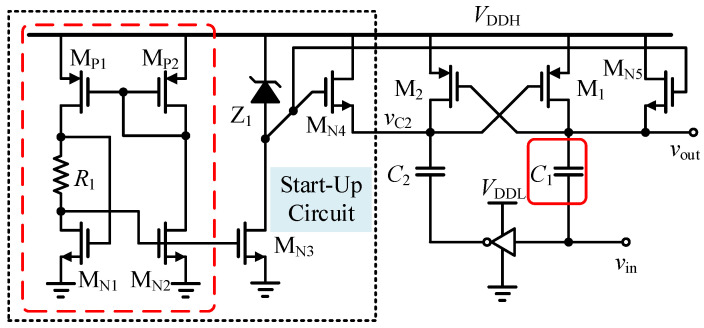
Level-shifting circuit with an added start-up circuit.

The staged trigger logic is implemented based on 5 V analog-level processing, whereas the LDMOS devices that control the gate-driving stage are referenced to the 24 V high-voltage supply rail. Conventional active level-shifting circuits based on cross-coupled P/N transistors are typically limited by the establishment time associated with positive-feedback latching, resulting in propagation delays of approximately 10–20 ns. To overcome this limitation, a capacitive-coupled near-zero-delay level-shifting architecture is adopted in this work, as shown in Figure 8.

This circuit abandons the slow static charge/discharge process associated with charge carriers and instead operates by exploiting the impedance difference in high-voltage micro-capacitors in the high- and low-frequency domains. However, this topology involves a potential risk: during the power-up initialization (startup) phase, if the coupling capacitor fails to establish the bias voltage drop synchronously, the thin gate oxide of the 5 V devices may suffer irreversible breakdown due to transient overvoltage. To address this issue, a self-biased charging protection network with Zener clamping is designed in this work. This module employs a high-voltage Zener diode to establish a clamping reference voltage and pre-charges the high-voltage plate of the coupling capacitor through a source follower. Simulation results confirm that, as the high-voltage rail gradually rises to 24 V, the plate voltage of the coupling capacitor automatically settles at a safe plateau of approximately 18.8 V, thereby ensuring that the *v*_GS_ of all subsequent transistors is reliably clamped within the safe limit of 5.2 V. The actual propagation delays of this circuit are only 660 ps and 540 ps for the rising and falling edges, respectively.

#### 3.3.2. High-Speed Comparator

The response triggering of the feedforward system relies on the comparator to determine the instant at which the externally scaled *v*_GS_ signal crosses the preset threshold *V*_SW1_. The comparator delay directly results in deviations in the stage-switching instant. Although dynamic clocked comparators exhibit very low power consumption, they require the system to provide an external clock at the GHz level, which introduces significant high-frequency digital crosstalk noise through substrate noise coupling. Therefore, such comparators are mainly suitable for specific applications such as analog-to-digital converters and are not appropriate for the standalone driver-chip application considered in this work.

**Figure 9:**
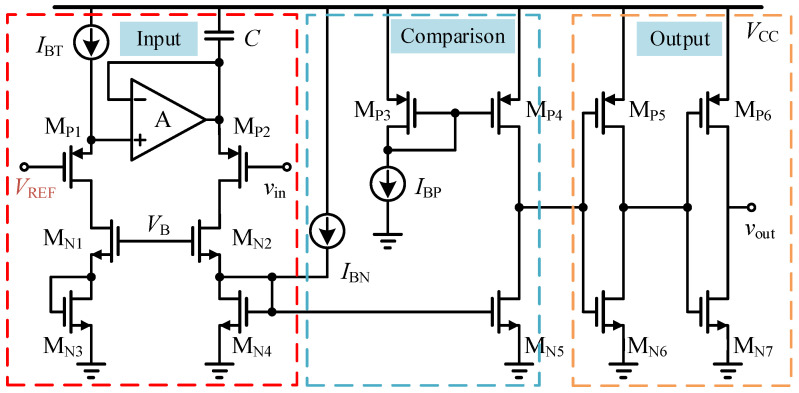
Simplified circuit of the high-speed comparator.

In this design, a fast-response static comparator with an asymmetrical differential input structure is proposed and implemented, as shown in Figure 9. Considering that the comparator reference threshold *V*_REF_ is a steady DC level, whereas the input signal *v*_in_ to be detected is a transient waveform at the nanosecond scale, the two input branches of the comparator are treated asymmetrically. Specifically, the *V*_REF_ signal is processed through a built-in preamplifier as a low-frequency bias network to compensate for the intrinsic threshold-voltage drift of the MOS transistors caused by process variation and junction-temperature fluctuation, while the input signal *v*_in_ to be detected is directly applied to the high-frequency branch. In addition, by means of a specially designed cross-biasing scheme, the core current decision transistors are maintained in either the saturation region or the linear region at the switching critical point. Small-signal analysis indicates that the gain–bandwidth product (GBW) of this structure is linearly related to the sum of the transconductances corresponding to the static bias current:

(22) $$GBW=\frac{g_{m}}{2\pi C}=\frac{1}{2\pi C}\sqrt{2\mu C_{\mathrm{OX}}\frac{W}{L}I_{DS}}.$$

By appropriately trading static bias current for response speed, the static comparator maintains the absolute decision delay within a narrow range of 1.76 ns to 2.26 ns under process-corner simulations across SS, TT, and FF when subjected to an input excitation with a steep 50 ns falling edge.

## 4. Validation of the Proposed Driver Chip

### 4.1. Chip Implementation and Test Platform

**Figure 10:**
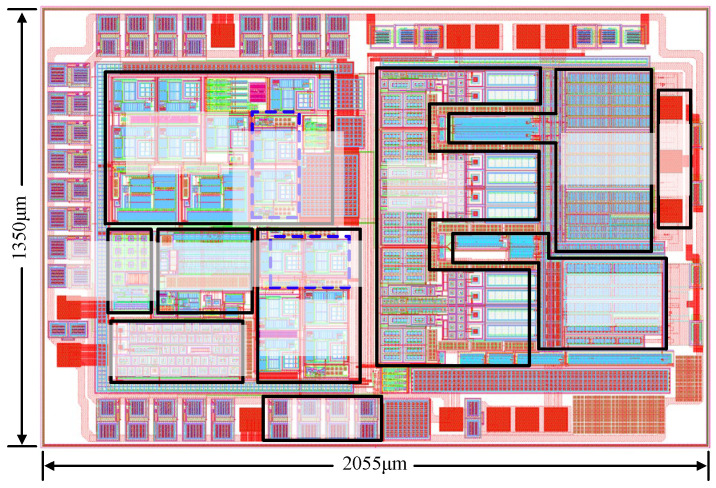
Overall layout of the driver chip.

The proposed driver chip was implemented and fabricated based on the HHGrace BD180GEZ8 process. The overall chip layout is shown in Figure 10, and the total chip area is approximately 2.774 mm^2^.

**Figure 11:**
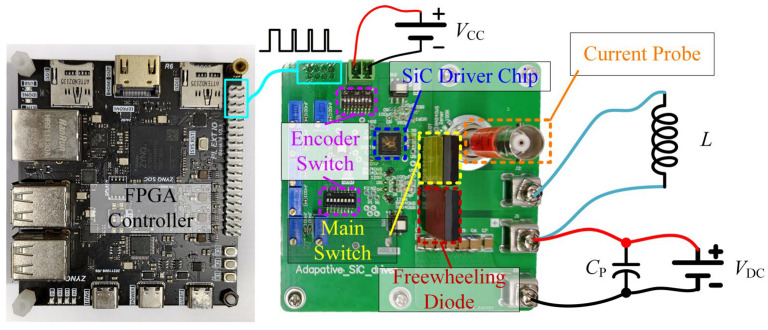
Driver chip test platform.

Figure 11 illustrates the test platform of the driver chip designed in this work, which mainly consists of an FPGA control board, a test board for the SiC adaptive driver chip, DC power supplies, and passive components. The FPGA is implemented using the Xilinx ZYNQ7010 platform and is employed to generate and control the driving pulses required for testing. The DC power supplies include the low-voltage supply *V*_CC_ for the driver chip and the high-voltage supply *V*_DC_ for the power loop. The test board is the core of the entire test platform, integrating the driver chip, power devices, and the associated peripheral circuits, and its layout is optimized to minimize the influence of parasitic parameters along critical signal paths. Both the main switch and the freewheeling device are implemented using the C3M0032120K SiC MOSFET manufactured by CREE. Considering that the proposed scheme adopts a cross-cycle control method, the Multi-Pulse Test (MPT) method is employed in the experiments to ensure that the control state of the driver chip can be stably established and effectively validated.

### 4.2. Experimental Results and Performance Evaluation

#### 4.2.1. Cross-Cycle Control Test

**Figure 12:**
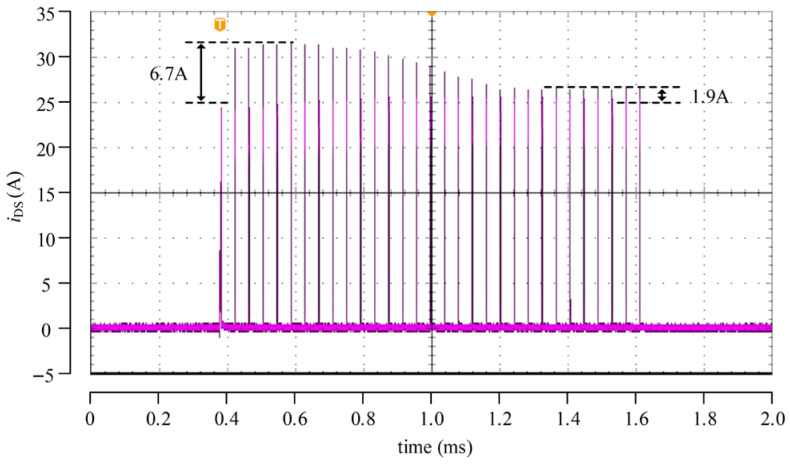
Cross-cycle control test waveforms.

Figure 12 presents the experimental results of the cross-cycle control of the SiC driver chip designed in this work under the test condition of 400 V/25 A. The waveform shown in the figure corresponds to the drain current *i*_DS_ of the main switching device measured using a current probe. Consistent with the simulation results, the current overshoot of *i*_DS_ is effectively suppressed after adjustment over multiple switching cycles and eventually converges to a stable state. This result directly verifies that the proposed cross-cycle control can update the switching point from one cycle to the next and gradually drive the turn-on process toward the desired operating condition.

#### 4.2.2. Adaptive Function Test

In the adaptive function test, considering the safety of high-voltage experiments, a 400 V DC bus voltage was adopted, and the adaptive capability of the driver chip was evaluated by varying the load current. To facilitate observation of the role of the three-stage control in different stages, the driving current in the second stage was intentionally reduced in the experiment so as to decrease the d*v*_DS_/d*t* during the voltage falling stage and prolong the duration of the second stage. As a result, the turn-on transient time and switching loss obtained in the subsequent tests are generally higher than the simulation results. The three-stage driving currents were set to 1.6 A, 0.2 A, and 2.3 A, respectively.

**Figure 13:**
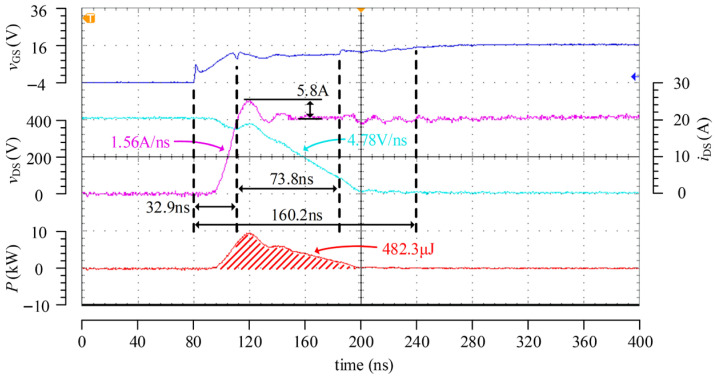
Turn-on test waveforms at 400 V/20 A.

Figure 13 presents the turn-on test results of the designed SiC adaptive active driver under the condition of 400 V/20 A, including the waveforms of *v*_GS_, *i*_DS_, *v*_DS_, and the calculated turn-on loss power. The results show that the total turn-on duration is approximately 160.2 ns, among which the first and second stages last 32.9 ns and 73.8 ns, respectively. Because the adopted device has a higher power rating and a larger input capacitance, the measured d*i*_DS_/d*t* is lower than the simulation result, with a value of only 1.56 A/ns. Meanwhile, the reduced driving current in the second stage decreases the d*v*_DS_/d*t* during the voltage falling stage to 4.78 V/ns, which in turn increases the turn-on loss to 482.3 μJ.

**Figure 14:**
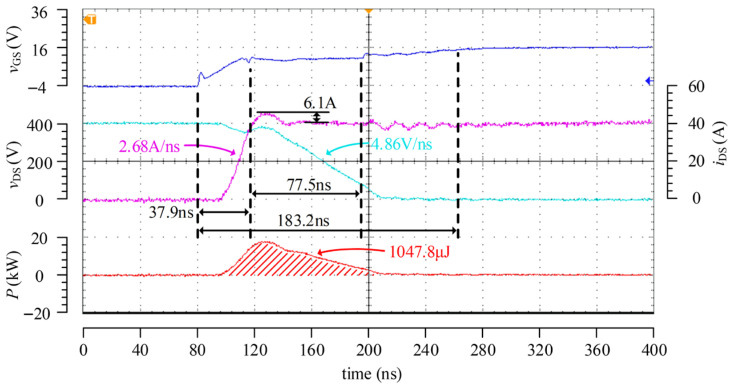
Turn-on test waveforms at 400 V/40 A.

Figure 14 presents the turn-on test results of the driver under the condition of 400 V/40 A. Compared with the case of 20 A, the duration of the first stage increases to 37.9 ns due to the elevated Miller plateau voltage, and the total turn-on time correspondingly increases to 183.2 ns, resulting in a turn-on loss of approximately 1048 μJ, which is significantly higher than that under the 20 A condition. Since the second-stage driving current remains the same under both operating conditions, the d*v*_DS_/d*t* level during the voltage falling stage is nearly identical, which is in good agreement with the theoretical analysis and simulation results. Although the d*i*_DS_/d*t* under the 40 A condition increases to 2.68 A/ns, the current overshoot rises only slightly to 6.1 A, indicating that the adopted optimized control strategy can effectively suppress overshoot.

As can be observed from the *i*_DS_ waveforms in Figure 13 and Figure 14, the designed driver chip can accurately identify the instant at which *i*_DS_ rises to the load current under different load conditions and implement the corresponding three-stage control, thereby verifying its expected adaptive regulation capability.

#### 4.2.3. Turn-Off Control Test

**Figure 15:**
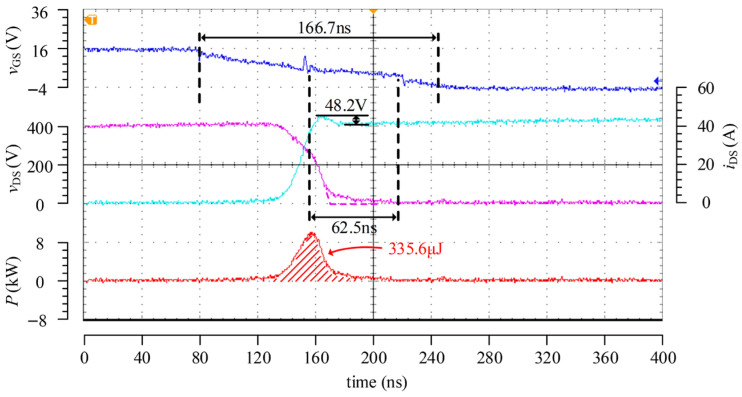
Turn-off test waveforms at 400 V/40 A.

Figure 15 presents the turn-off test results of the driver under the condition of 400 V/40 A. Since the three-stage control during the turn-off process in this work does not possess adaptive capability, this part mainly verifies its three-stage control function. In the test, the peak turn-off currents of the three stages were set to 0.8 A, 0.2 A, and 1.5 A, respectively. The results show that the turn-off loss is 335.6 μJ, which is significantly lower than the turn-on loss, thereby validating the theoretical analysis that the turn-off loss is relatively low. In the experiment, the duration of the second stage reached 62.5 ns, which is higher than the expected value. This is mainly because the switching instant based on the divided *v*_DS_ feedback signal is difficult to determine accurately when *v*_DS_ approaches a steady state, resulting in a slight delay in the switching between the second and third stages. Consequently, after *v*_DS_ rises to the DC bus voltage, the device is not turned off completely immediately, leading to a certain degree of current tailing and introducing additional turn-off loss.

### 4.3. Comparative Analysis with the Conventional Driving Method

In this section, the designed SiC adaptive active driver chip is compared with a conventional passive driver to evaluate its optimization effects on the turn-on and turn-off processes. The reference driver selected for comparison is the Si8271 passive driver chip manufactured by Skyworks, whose driving capability is adjusted by varying the external gate resistor.

**Figure 16:**
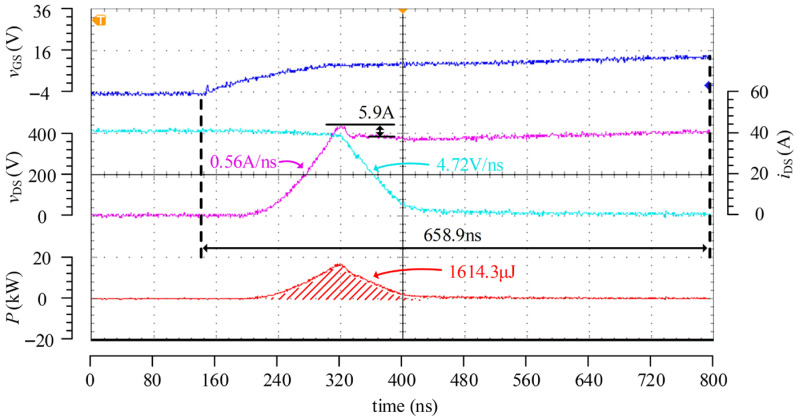
Turn-on test waveforms of the passive driver at 400 V/40 A.

Figure 16 presents the turn-on test waveforms of the passive driver under the condition of 400 V/40 A, where the turn-on gate resistance is set to 36 Ω. The results show that, under the same test condition, the d*v*_DS_/d*t* of the passive driver is close to that of the proposed driver, both being approximately 4.8 V/ns. By comparing Figure 14 and Figure 16, it can be seen that, under a similar d*v*_DS_/d*t* level, the turn-on loss of the proposed driver is 1047.8 μJ, whereas that of the passive driver is 1614.3 μJ, corresponding to an improvement of 35.1%. Meanwhile, the turn-on transient time of the passive driver exceeds 650 ns, whereas that of the proposed driver is only 183.2 ns, indicating that the latter is more suitable for the high-frequency operation of SiC MOSFETs. In addition, the d*i*_DS_/d*t* of the proposed driver during turn-on is 2.68 A/ns, with a current overshoot of approximately 6.1 A. In contrast, the d*i*_DS_/d*t* of the passive driver is only 0.56 A/ns, while the current overshoot still reaches 5.9 A. This indicates that the adaptive active driver chip designed in this work can effectively suppress current overshoot while achieving a significantly faster turn-on speed.

**Figure 17:**
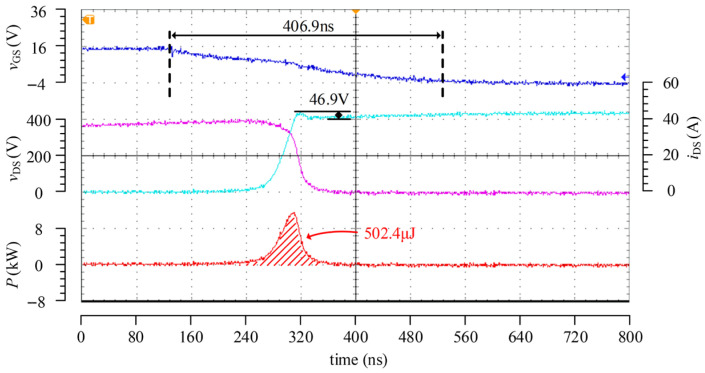
Turn-off test waveforms of the passive driver at 400 V/40 A.

The turn-off process of the passive driver was also tested under the condition of 400 V/40 A, and the corresponding waveforms are shown in Figure 17, where the turn-off gate resistance was set to 30 Ω. As can be observed from Figure 15 and Figure 17, the drain–source voltage overshoot during turn-off is approximately 50 V for both driving methods. Under a similar overshoot level, the turn-off loss of the passive driver is about 502.4 μJ, whereas that of the proposed active driver is 335.6 μJ, corresponding to a reduction of approximately 33.2%. Even though a certain deviation exists in the switching from the second stage to the third stage, the proposed driving scheme still exhibits a lower turn-off loss, thereby validating its optimization effect on the turn-off process.

## 5. Conclusions

This study addresses the trade-off between switching loss and electromagnetic interference in SiC MOSFETs under high-frequency and high-voltage operating conditions by proposing an adaptive active gate driver chip based on cross-cycle feedforward control, fabricated in the HHGrace BD180GEZ8 process. By using the Miller plateau voltage as the adaptive sensing quantity and combining it with a three-stage driving strategy, the proposed scheme applies stronger drive at the beginning and end of switching to shorten the transient interval, while reducing the gate current during the EMI-sensitive stage to balance switching speed and overshoot suppression. Multi-pulse test results show that, under the same d*v*/d*t* and voltage-overshoot conditions, the proposed driver shortens the switching transient to 183.2 ns and reduces turn-on and turn-off dynamic losses by 35.1% and 33.2%, respectively, compared with a conventional passive driver. In addition, the chip maintains favorable overshoot suppression and dynamic regulation under variations in load current, and the cross-cycle mechanism remains effective under temperature variation. Overall, the proposed highly integrated driver architecture provides an effective solution for improving the driving performance of SiC MOSFETs in high-efficiency and high-power-density power conversion systems.

## Data Availability

Data are contained within the article.
